# Risk factors and treatments for brain metastasis in patients with adenocarcinoma of the lung: a retrospective analysis of 373 patients

**DOI:** 10.1186/s41016-018-0113-z

**Published:** 2018-04-26

**Authors:** Bo Li, Zhaoxia Dai, Shuai Liu, Xuenan Gu, Yanwei Liu, Xiaoguang Qiu

**Affiliations:** 1 Department of Radiation Oncology, Beijing Tiantan Hospital affiliated to Capital Medical University, No. 6, Tiantan Xili, Dongcheng District, Beijing, 100050 China; 2 Department of Medical Oncology, The Second Hospital of Dalian Medical University, No. 467, Zhongshan Road, Shahekou District, Dalian, 116027 Liaoning Province China

**Keywords:** Non-small cell lung cancer, Risk factor, Adenocarcinoma, Brain metastasis, Epidermal growth factor receptor

## Abstract

**Background:**

Risk factors and treatments for brain metastasis (BM) in patients with adenocarcinoma have not been fully profiled in previous studies because of the enrolment of patients with tumours of mixed histology. Thus, we specifically addressed the issue in patients with adenocarcinoma.

**Methods:**

Clinical data for 373 patients with pathologically confirmed adenocarcinoma were studied retrospectively. Factors including age (≤60 vs. > 60), gender (male vs. female), stage at diagnosis, T status (T1–2 vs. T3–4), N status (N0–1 vs. N2–3), epidermal growth factor receptor (EGFR) mutation status (wild-type vs. mutant) and smoking status (never vs. current) were analyzed.

**Results:**

In multivariate analysis, age (*P* = 0.006) and N status (*P* = 0.041) were independent risk factors for BM. In patients with BM, adding systemic therapy to local therapy improved median post-brain-metastasis survival (mPBMS) (*P* = 0.02). However, if stratification was conducted according to the recursive partitioning analysis (RPA) classification or graded prognostic assessment (GPA) scoring, only patients in RPA class II (*P* = 0.020) or with GPA score 1.5-2.5 (*P* = 0.032) could benefit from local plus systemic therapy. Those who received both pemetrexed and tyrosine kinase inhibitors (TKIs) as systemic therapies had a longer mPBMS than those who received TKIs alone, regardless of whether local therapy was applied. In patients with EGFR-sensitive mutations, TKIs therapy led to a longer mPBMS than conventional chemotherapy (*P* = 0.002).

**Conclusions:**

Adenocarcinoma patients who were younger than 60 years of age and those with N2–3 disease have a significantly higher risk of BM. The addition of systemic therapy to local therapy can significantly prolong mPBMS, but the survival benefit confined in certain populations. Patients with opportunity to receive both pemetrexed and TKIs had the longest mPBMS.

## Background

Adenocarcinoma accounts for approximately 40% of all cases of non-small cell lung cancer (NSCLC), and its incidence continues to rise [1]. Clinically, it is characterized by aggressive course, rapid progression and early distant metastases [2]. The brain is one of the most common sites of distant metastasis. Prophylactic cranial irradiation (PCI) has been the standard of care for patients with small cell lung cancer (SCLC), which has been shown not only to decrease the incidence of BM but also to improve long-term survival [3–6]. Similar studies have been conducted in patients with NSCLC. Unfortunately, although the incidence of BM was shown to be decreased by PCI, no survival advantage was observed [7, 8]. One likely explanation for this is the heterogeneous risk of BM across pathological subtypes. Many studies have found that the incidence of BM in patients with adenocarcinoma is significantly higher than that in other subtypes, such as squamous cell carcinoma (SCC) [2, 9–11]. However, the risk factors for BM in patients with adenocarcinoma have not been fully profiled in previous studies because of the enrolment of patients with tumours of mixed histology. Thus, comprehensive studies are needed to specifically address the clinical features of BM in patients with adenocarcinoma, as they may provide useful information so that future studies of BM prevention in NSCLC may be tailored.

For patients with BM, local therapies such as whole brain radiation therapy (WBRT), stereotactic radiosurgery (SRS), and surgical resection are the standard of care. However, because most patients die from systemic disease rather than from intracranial failure, using systemic therapy as part of post-BM treatment has been advocated by many physicians. In addition to third-generation agents, pemetrexed and tyrosine kinase inhibitors (TKIs) have demonstrated superior efficacy in terms of response rate and median overall survival (OS) in patients with adenocarcinoma [12–14]. However, few studies have compared the efficacy of the different systemic regimens mentioned above in patients with adenocarcinoma and BM. To explore the risk factors for BM and the optimal post-BM treatment strategies for patients with adenocarcinoma, we conducted a retrospective study that included 373 patients.

## Methods

### Patients and ethics

The inclusion criteria were patients with pathologically confirmed adenocarcinoma and complete medical records. In practice, dozens of patients with tumours of non-squamous cell carcinoma histology, especially those with known EGFR status, were also included. Two experienced pathologists from two hospitals reviewed all specimens separately and common consensus was reached. The study was reviewed and approved by the institutional review boards and ethics committees of Beijing Tiantan Hospital affiliated to Capital Medical University and The Second Hospital of Dalian Medical University. All patients provided informed consent according to the Declaration of Helsinki.

### Treatment strategy and follow-up

A central consultation board coordinated treatment strategy of two hospitals. At the time of diagnosis, baseline assessments of medical history, physical examination, radiographic examinations, biochemistry and blood routine test was conducted for all patients. In terms of treatment strategy, platinum-doublets were recommended post-operatively for patients with operable stage IB to IIIA disease. In fact, this has been the routine recommendation since 2006, when concrete evidence was first obtained from studies on adjuvant chemotherapy. For patients with advanced disease (stage IIIB to IV disease), systemic therapy was prior option. In contrast, for patients with EGFR mutations or advanced/relapsed disease, TKIs were considered. Baseline assessments were repeated at the completion of the planned therapy and were then repeated every 3 months for the first 2 years, and every 6 months for the next 3 years.

### BM screening and treatment

Enhanced-contrast MRI was used for BM screening if there were no contraindications. Otherwise, enhanced-contrast CT was used. At the time of diagnosis, MRI was routinely performed for all patients. During the follow-up period, MRI was conducted every 3-6 months. If symptoms of CNS metastasis were present during the follow-up interval, MRI was performed immediately. Generally, SRS was reserved for patients with documented 1-3 BM lesions. Otherwise, whole-brain radiotherapy was considered. After the completion of radiotherapy, systemic therapy was routinely recommended.

### EGFR mutation testing

Paraffin-embedded tissue sections were used for EGFR mutations testing. Briefly, an amplification refractory mutation system was employed for EGFR mutation detection with an ADx EGFR Mutations Detection Kit (Amoy Diagnostics, Xiamen, China). QIAamp DNA Mini Kit (Qiagen Inc., Valencia, CA, USA) was used for DNA extraction. This assay was performed in an ABI 7500 (Applied Biosystems, Foster City, CA, USA) real-time polymerase chain reaction system according to the manufacturer’s protocol.

### Statistical analysis

Data analysis was conducted by using IBM SPSS Statistics 19.0 software. The Kaplan–Meier method was employed to calculate the median survival. Brain-metastasis-free survival (BMFS) was determined from diagnosis to the date at which BM was documented radiographically. Post-brain-metastasis survival (PBMS) was calculated from the date of documented BM to the date of death or the last follow-up visit. The log-rank test was used to compare the survival curves. Cox regression was used for the multivariate analysis, and the chi-squared test was employed to compare the incidence of BM among patients with different risk factors.

## Results

### Patient characteristics

Altogether, the clinical data for 373 consecutive patients who were diagnosed between September 2006 and October 2014 were selected from the database (214 patients from the database of Beijing Tiantan Hospital affiliated to Capital Medical University, and 159 from the database of The Second Hospital of Dalian Medical University). The characteristics of the patients are detailed in Table 1.

**Table 1:** Patients’ characteristics and incidence of BM according to risk factors

Variables	No.	%	No. of BM	Incidence of BM(%)	*P* value
Gender					0.33
Male	209	56	104	49.8	
Female	164	44	87	53.0	
Age					0.007
≤ 60	200	53.6	120	60.0	
> 60	173	46.4	71	41.0	
Stage					0.001
I/II	78	26.4	26	33.3	
III/IV	295	73.6	165	55.9	
T status					0.67
T1-2	251	67.3	131	52.2	
T3-4	122	32.7	60	49.2	
N status					0.024
N0-1	159	42.6	67	42.1	
N2-3	214	57.4	124	57.9	
EGFR status(*N* = 100)					0.48
Wild-type	49	49.0	22	44.9	
Mutation	51	51.0	20	39.2	
Histology					< 0.001
Adenocarcinoma	345	92.5	184	53.3	
Non-adenocarcinoma	28	7.5	7	25.0	
Smoking status					0.39
Never	239	64.1	124	55.6	
Current	134	35.9	67	50.0	

*Abbreviations*: *BM* brain metatasis, *EGFR* epidermal growth factor receptor, *BAC* Bronchioloalveolar carcinoma

### Risk factors for brain metastasis

Patients younger than 60 years of age (*p* = 0.007), those with advanced stage disease (*p* = 0.001) and those with N2-3 disease at diagnosis (*p* = 0.024) had a significantly higher incidence of BM than their counterparts. Altogether, 100 patients with known EGFR status whose details were discussed in our previous report, were enrolled in our study [15]. In short, the mutation rate was 51%. Among those with a known EGFR status, the highest incidence of BM was found in patients with mutations at exon 19. However, the difference in the incidence of BM was not significant between those with wild-type EGFR (44.9%, 22/49) and patients with EGFR mutations (39.2%, 20/51) (*p* = 0.48). In all, 92.5% of patients in our cohort had histology that was consistent with adenocarcinoma. The incidence of BM was 53.3%, which was significantly higher than that of patients with histology that was not indicative of adenocarcinoma (*p* < 0.001). However, other factors such as gender (*p* = 0.33), smoking status (*p* = 0.39) and T status (*p* = 0.67) were not correlated with the incidence of BM (Table 1).

**Fig. 1:**
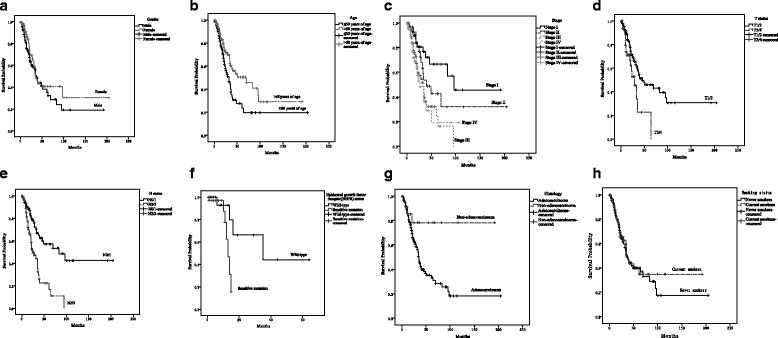
Brain-metastasis-free survival analysis according to various risk factors. **a** Gender (male vs female, *p* = 0.25), **b** Age (≤60 vs > 60, *p* = 0.006), **c** Stage at diagnosis (*p* = 0.002), **d** T status at diagnosis (T1/2 vs T3/4, *p* = 0.009), **e** N status at diagnosis (N0/1 vs N2/3, *p* < 0.001), **f** EGFR status (wild-type vs sensitive mutation, *p* = 0.018), **g** Histology (adenocarcinoma vs non-adenocarcinoma, *p* = 0.02), **h** Smoking status (never smoker vs current smoker, *p* = 0.69)

In addition to the incidence of BM, BMFS was employed to evaluate the risk of BM for a given patient. As is shown, younger patients had a significantly shorter mBMFS than older patients (*p* = 0.006). Otherwise, patients with mutation at exon 19/21/and dual mutation (EGFR-sensitive mutations) (*p* = 0.018), adenocarcinoma histology (*p* = 0.02), advanced stage (*p* = 0.002), advanced T status (*p* = 0.009) and advanced N status (*p* < 0.001) at diagnosis had a significantly shorter mBMFS than their counterparts. Moreover, the mBMFS in patients of different genders (*p* = 0.25) and in patients with different smoking statuses (*p* = 0.69) was comparable. A multivariate analysis found that only age (*p* = 0.006) and N status (*p* = 0.041) were independent risk factors for BM (Table 2, Fig. 1a-h).

**Table 2:** BMFS comparison according to risk factors

	Univariate			Multivariate		
Variables	BMFS(month)	95%CI	*P* value	*P* value	RR	95%CI
Gender			0.25	0.072	0.293	0.077-1.114
Male	37	31.2-42.7				
Female	36	30.1-41.8				
Age			0.006	0.006	0.201	0.065-0.624
≤ 60	32	22.9-41.0				
> 60	70	27.9-112.0				
Stage			< 0.001	0.68	1.166	0.554-2.455
I/II	114	47.3-180.6				
III/IV	27	20.7-33.2				
T status			0.009	0.43	1.648	0.469-5.791
T1-2	46	19.0-72.9				
T3-4	29	20.1-37.8				
N status			< 0.001	0.041	2.891	1.045-7.996
N0-1	83	31.5-134.4				
N2-3	25	15.5-34.4				
EGFR status			0.018	0.57	1.350	0.478-3.818
Wild-type	35	0-70.6				
Sensitive mutation	13	10.6-15.3				
Histology			0.02	0.38	0.402	0.051-3.155
Adenocarcinoma	35	30.9-39.0				
Non- Adenocarcinoma	Not reached					
Smoking status			0.69	0.18	0.418	0.115-1.518
Never	36	29.2-42.7				
Current	37	31.3-42.7				

Stage was not analyzed simultaneously with T/N status in multivariate analysis

*Abbreviations*: *BMFS* brain-metastasis-free survival, *EGFR* epidermal growth factor receptor

### Treatments after BM

**Fig. 2:**
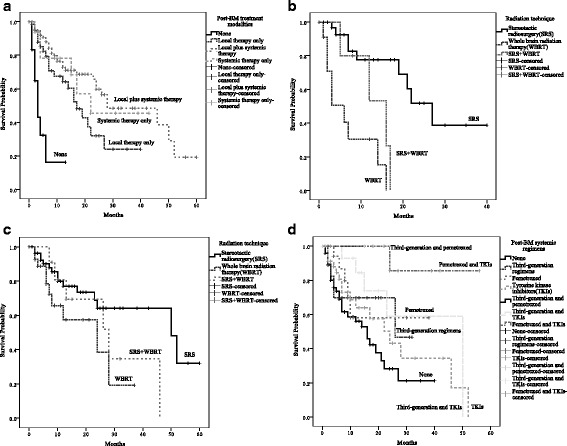
Post-brain-metastasis survival (PBMS) analysis according to treatment regimens. **a** PBMS comparison according to post-BM treatment strategy (*p* < 0.001), **b** PBMS comparison according to radiation technique in patients receiving local therapy only (*p* < 0.001), **c** PBMS comparison according to radiation technique in patients receiving local and systemic therapy (*p* = 0.076), **d** PBMS comparison according to systemic regimens (*p* = 0.009)

In patients with documented BM, the treatments that were given after BM was detected are summarized in Table 3. The mPBMS for the recursive partitioning analysis (RPA) classes I–III were 46 months, 27 months and 5 months, respectively (*p* < 0.001). The mPBMS according to the graded prognostic assessment (GPA) score were as follows: GPA 0–1, 9 months; GPA 1.5–2.5, 28 months; GPA 3, 27 months; and GPA 3.5–4.0, not reached (*p* < 0.001). In terms of treatment modality, the mPBMS was only 3 months for those who received no treatment after the detection of BM, 17 months for patients who received local therapy only, 22 months for patients who received systemic therapy only and 28 months for patients who received both (*p* < 0.001) (Fig. 2a). The difference in the values of the mPBMS was significant between those who received local plus systemic therapy and those who received local therapy only (*p* = 0.020). However, if stratification was conducted according RPA or GPA, only patients in RPA class II (*p* = 0.020) or with GPA score 1.5-2.5 (*p* = 0.032) could benefit from local plus systemic therapy.

**Table 3:** Characteristics of patients with BM

Variables	No.	%
Number of BM		
1	97	50.8
2-3	39	20.4
> 3	55	28.8
Presence of systemic metastasis at the time of BM		
Yes	161	84.3
No	30	15.7
Treatments after BM		
None	14	7.3
Local therapy only	63	33.0
Local+systemic therapy	98	51.3
systemic therapy only	16	8.4
Local therapy (*n* = 161)		
SRS	105(9)^a^	65.2
WBRT	40(1)	24.8
SRS + WBRT	16(2)	10.0
Systemic therapy regimens(*n* = 114)		
Third-generation regimens	20	17.5
Pemetrexed	19	16.7
TKIs	37	32.5
Third-generation+pemetrexed	4	3.5
Third-generation+TKIs	19	16.7
Pemetrexed+TKIs	15	13.1

*Abbreviations*: *BM* brain metatasis, *RPA* Recursive partitioning analysis, *GPA* graded prognostic assessment, *WBRT* whole brain radiation therapy, *SRS* stereotactic radiosurgery, *TKIs* tyrosine kinase inhibitors

^a^Number in the blanket was number of patients who received surgery for brain lesions

For patients who received local therapy only after the detection of BM, those who received SRS had a significantly longer mPBMS than those who received WBRT (27 months vs 6 months, *p* < 0.001) (Fig. 2b). If stratification was conducted according to the number of intracranial lesions, SRS could significantly improve the mPBMS of patients with 1-3 lesions compared with WBRT (27 months vs 6 months, *p* = 0.008) or WBRT plus SRS (27 months vs 12 months, *p* < 0.001). For patients with four or more BM lesions, those who received WBRT plus SRS had a longer mPBMS (17 months) than those who received SRS (9 months) or WBRT alone (3 months), but these differences were not significant (*p* = 0.31).

For the patients who received local plus systemic therapy after the detection of BM, the mPBMS was 50 months for those who received SRS, 24 months for those who received WBRT and 28 months for those who received SRS and WBRT. The difference in the mPBMS was significant between those who received SRS and those who received WBRT (*p* = 0.038), but was comparable between those who received WBRT and those who received WBRT and SRS (*p* = 0.27) (Fig. 2c). Moreover, unlike the patients who received local therapy only, in patients with 1-3 or 4 or more intracranial lesions, the differences in the PBMS among patients who received one of the three radiation modalities were not significant.

In terms of systemic therapy after BM, we grouped the patients according to the therapy regimens. According to the survival analysis, those patients who received pemetrexed and TKIs had a significantly longer mPBMS than those who received TKIs only (not reached vs 24 months, *p* = 0.009). Furthermore, compared with third-generation regimens (*p* = 0.071), third-generation regimens/TKIs (*p* = 0.084) and pemetrexed only (*p* = 0.075), the advantage of pemetrexed and TKIs in terms of mPBMS reached marginal significance. Even in patients who received local and systemic therapies, those who received pemetrexed and TKIs had a significantly longer mPBMS than those who received TKIs only (not reached vs 26 months, *p* = 0.031). However, the differences between other regimens did not reach statistical significance. In patients with EGFR-sensitive mutations, those who took TKIs as part of a post-BM systemic therapy regimen had a significantly longer mPBMS than those who were treated with conventional chemotherapy (not reached vs 9 months, *p* = 0.002). However, in patients with wild-type EGFR, the PBMS was longer in those who received conventional chemotherapy than in those who received TKIs, but the difference did not reach statistical significance (*p* = 0.104) (Fig. 2d).

## Discussion

Studies of PCI in patients with NSCLC have failed to reproduce the results obtained in patients with SCLC [7, 8]. The reason for this may lie in the different biological behaviour of the malignant cells in these two diseases. Firstly, the incidence of BM in patients with SCLC ranged from 40 to 58% [3, 4, 6], which is higher than that in NSCLC patients (ranged from 18 to 38%) [7–10]. Secondly, NSCLC comprises many histological subtypes, each with a different incidence of BM [2, 11, 16, 17]. Data from our study showed that in patients with adenocarcinoma, which is the main subtype of non-SCC histology, the incidence of BM was 53.3%; this was significantly higher than in patients in our cohort with other non-SCC subtypes. Additionally, this incidence is similar to that which has been reported in patients with SCLC. Furthermore, a risk factor analysis also found that the incidence of BM was significantly higher in patients in specific subgroups. Based on these results, it is reasonable that adenocarcinoma patients, especially those with risk factors, should be potential candidates for future studies of BM prevention in the setting of NSCLC.

In addition to incidence, the interval from the diagnosis to the occurrence of BM is another parameter that we thought was useful in the evaluation of a patient’s risk of BM. As shown in our study, patients with specific risk factors had significantly shorter mBMFS than their counterparts. The mBMFS of patients with SCLC is unknown because most studies have employed cumulative incidence as an evaluation parameter. In a randomized study that was conducted in patients with extensive stage SCLC, for the patients who received PCI, the median DFS and median OS were 14.7 weeks and 6.7 months, respectively [6]. While in our cohort, the mBMFS of patients with metastatic disease at the time of diagnosis was 27 months. This is even longer than the median OS of patients with extensive stage SCLC, as mentioned above. Thus, it is suggested that, the occurrence of BM is an early event for patients with SCC but a late event for patients with adenocarcinoma. Considering the results of studies of PCI in cases of NSCLC, it is hypothesized that PCI, as a local therapy, exerts a powerful short-term effect, which involves a reduction in the cumulative incidence of BM. However, as time passes, the long-term effect of PCI on survival decreases, and it is replaced by other treatment modalities. Thus, the determination of the proper strategies of BM prevention in patients with NSCLC requires further investigation.

Similar to other studies, patients with BM without treatment in our cohort had the worst prognosis [18–20]. While for those with the opportunity to receive some type of treatment, the mPBMS was significantly prolonged. Radiotherapy is still the preferred option for patients with BM due to its ability to relieve neurological symptoms and its potential role in prognostic improvement in select populations [20–23]. However, in terms of systemic therapy, there may be hesitation, because theoretically it is hard for most water-soluble agents to penetrate the blood-brain barrier (BBB). Otherwise, patients’ Karnofsky performance status (KPS) at the time of BM is another concern. It is thought that the BBB might be disrupted by the time that BM is detected or after the patients have been treated with radiotherapy [24, 25]; this concept is supported by the objective response of intracranial lesions after systemic therapy [26, 27]. Data from our studies indicated that systemic therapy not only improved the mPBMS as an independent treatment modality but also was associated with a greater survival benefit for patients with BM when combined with local therapy. However, further analysis found that survival benefit confined in patients in RPA class II or with GPA score 1.5-2.5. Given that BM is a type of haematogenous metastasis, it is reasonable that systemic therapy be considered as part of post-BM therapy, but it should be reserved in certain populations.

In terms of local therapy, SRS and WBRT are both common non-invasive techniques. Generally speaking, SRS has a satisfactory local control rate if intracranial lesions are less than 4 cm in diameter and no more than 4 in number [28]. Some studies have explored the possibility of the combination of WBRT and SRS in patients with a limited number of intracranial lesions [29, 30]. However, the results are conflicting. As shown in our study, in patients with 1-3 intracranial lesions, SRS was associated with the longest mPBMS, regardless of whether systemic therapy was applied. Thus, by far, SRS still should be priority for patents with a limited number of intracranial lesions.

Pemetrexed and TKIs have both demonstrated an active role in the treatment of adenocarcinoma. Pemetrexed established its role based on the results of the JMDB study, in which a subgroup analysis demonstrated superior efficacy of pemetrexed in patients with adenocarcinoma histology [13]. Furthermore, an objective response of intracranial lesions has been observed in patients with BM who received pemetrexed as a first-line or a second-line therapy [31–33]. Owing to the higher prevalence of EGFR mutations, TKIs have shown a dramatic efficacy in patients with adenocarcinoma [12, 14]. Additionally, several clinical studies have reported their efficacy in patients with NSCLC and BM with an intracranial response that ranges from 26.6 to 82.9% when used as single agents [34–37] and 63–86% when combined with WBRT [34, 38, 39]. As shown in our study, those who were treated with post-BM systemic therapies that include pemetrexed and TKIs had the longest mPBMS. In addition, the difference in the survival of patients who received these therapies reached statistical significance compared with patients who received TKIs alone. Even in patients who received local therapy as a part of post-BM treatment, the significance remains between pemetrexed/TKIs and TKIs alone. Unfortunately, the difference in the mPBMS between patients who received pemetrexed/TKIs and patients who received regimens other than TKIs alone did not reach statistical significance. We attribute this to smaller samples in each systemic regimen subgroup and a relative short follow-up period after the detection of BM. Moreover, it seems that EGFR status is still useful to tailor the treatment strategies of TKIs in BM patients. It is shown that patients with mutation at exon 19/21/and dual mutation benefited more from post-BM treatment of TKIs than from conventional chemotherapy, although the difference in the PBMS between treatment with TKIs and treatment with conventional chemotherapy in patients with wild-type EGFR did not reach statistical significance.

## Conclusions

Overall, this is a retrospective study that was conducted exclusively in adenocarcinoma patients of the lung in order to explore the risk factors and treatments for BM. Based on our findings, patients with adenocarcinoma histology, especially those with risk factors of BM, should be the candidates for future studies concerning BM prevention in NSCLC setting. After the occurrence of BM, the addition of systemic therapy to local therapy can significantly prolong mPBMS. But the survival benefit confined in certain populations. Pemetrexed and TKIs are optimal systemic regimens in adenocarcinoma patients with BM due to their potential prognosis-improving ability.

## Abbreviations

BBB: Blood-brain barrier
BM: Brain metastasis
EGFR: Epidermal growth factor receptor
GPA: Graded prognostic assessment
KPS: Karnofsky performance status
mBMFS: Median brain-metastasis-free survival
mPBMS: Median post-brain-metastasis survival
NSCLC: Non-small cell lung cancer
OS: Overall survival
PCI: Prophylactic cranial irradiation
RPA: Recursive partitioning analysis
SCLC: Small cell lung cancer
SRS: Stereotactic radiosurgery
TKI: Tyrosine kinase inhibitor
WBRT: Whole brain radiation therapy
